# Pestivirus 2024: Special Issue Editorial

**DOI:** 10.3390/v17121621

**Published:** 2025-12-16

**Authors:** Benjamin J. Lamp, Christiane M. Riedel

**Affiliations:** 1 Institute of Virology, Justus-Liebig-Universität Gießen, Schubertstrasse 81 (BFS), 35392 Giessen, Germany; 2 CIRI-Centre International de Recherche en Infectiologie, Université Claude Bernard Lyon 1, Inserm, U1111, CNRS, UMR5308, ENS Lyon, 46 Allee d’Italie, 69007 Lyon, France; christiane.riedel@ens-lyon.fr

## 1. Introduction

Pestiviruses are well known in the veterinary field and include some of the most economically significant pathogens of ungulates, such as classical swine fever virus (CSFV) and bovine viral diarrhea virus (BVDV). In recent years, several related viruses have been discovered in new hosts, including cetaceans, rodents, and pangolins. Although decades of research have clarified many aspects of pestivirus infections, replication, regulation, and epidemiology, the field of pestivirus virology remains dynamic. Recent progress has focused particularly on cellular receptors and the mechanisms of viral entry. At the same time, efforts to eradicate pestiviruses from domestic animal populations and to understand their spread and epidemiology remain both scientifically and economically important. This Special Issue highlights these developments, presenting advances in basic research, diagnostics, and epidemiology. Yet significant knowledge gaps persist, and each new finding raises new scientific questions.

## 2. Content of This Special Issue

Aitkenhead et al. [1] presented the bovine CD46 ectodomain structure, showing four short-consensus-repeat (SCR) domains, with an angled SCR4 that may influence pestivirus receptor interactions. Buckley et al. [2] monitored atypical porcine pestivirus (APPV) infection from birth to market and showed that pigs from congenital tremor (CT) negative litters suffer from viremia and seroconvert, while CT-positive pigs usually remain persistently PCR-positive with limited antibody responses. Falkenberg et al. [3] used a multivariate antigenic analysis to demonstrate substantial diversity among BVDV-1b isolates and variable relatedness to vaccine strains, indicating possible gaps in current vaccine coverage. Hinojosa et al. [4] identified virulence determinants of CSFV by comparing related high- and low-virulent strains, showing that mutations across E2, NS5A/NS5B, and the 3′-UTR collectively drive attenuation or virulence. Sozzi et al. [5] reported the isolation of Tunisian sheep-like pestivirus (TSV, *Pestivirus N*) from persistently infected sheep in Northern Italy and linked its circulation to international animal movement. Tong et al. [6] evaluated four engineered CSF vaccine candidates in pregnant sows, of which two are safe, not vertically transmitted, and suitable for DIVA differentiation using marker-based ELISA methods. Huynh et al. [7] showed that two chimeric CSF marker vaccines, namely vGPE−/PAPeV-E^rns^ and vGPE−/PhoPeV-E^rns^, remain stably attenuated during serial passaging in pigs, with the PAPeV-E^rns^ chimera clearing faster following pig inoculation. Both vaccines displayed favorable interferon-inducing profiles, supporting their safety. Yin et al. [8] demonstrated that the natural compound bergamottin inhibits BVDV replication in cells and mice. Kalaiyarasu et al. [9] reported the detection of border disease virus 3 (BDV-3) in a persistently infected lamb from a migratory flock in India, revealing high seroprevalence, genetic variation, and links to Chinese BDV-3 strains, emphasizing risks associated with seasonal animal movement. Chen et al. [10] found that pestiviruses modulate the host factor ADAM17 in a species-specific manner, with classical pestiviruses reducing surface levels of ADAM17, while Linda virus and APPV did not, suggesting roles in entry and linking superinfection exclusion to ADAM17 interactions. Geranio et al. [11] compared superinfection dynamics across pestivirus species and showed similar exclusion patterns between classical pestivirus species. However, an enhanced susceptibility was found after APPV pre-infection, consistent with APPV using ADAM17-independent entry routes. Beilleau et al. [12] showed that the viral RNase E^rns^ from BVDV and Bungowannah virus efficiently suppresses dsRNA-induced interferon in differentiated airway epithelial cells, supporting a role in respiratory immunosuppression and persistent infection. Anoyatbekova et al. [13] identified APPV in multiple Russian regions with diverse phylogenetic clusters related to European, American, and Korean strains, confirming its circulation since at least 2020 (and demonstrating successful isolation of one strain in cell culture). Coronado et al. [14] profiled gene expression in porcine bone-marrow antigen-presenting cells (APC) infected with low- or highly virulent CSFVs and found that the attenuated strain induces ISGs and immune-activating pathways, whereas the lethal strain triggers checkpoint molecules, inflammatory mediators, and suppression of T-cell differentiation pathways, providing mechanistic clues to CSF pathogenicity and virulence.

## 3. Conclusions

The findings presented in this Special Issue demonstrate exciting advances in our understanding of the biology, epidemiology, and vaccinology of pestiviruses. We hope the collection offered here provides a valuable snapshot of current progress and a platform that both summarizes recent findings and inspires further discoveries.
